# Interaction of Atmospheric-Pressure Air Microplasmas with Amino Acids as Fundamental Processes in Aqueous Solution

**DOI:** 10.1371/journal.pone.0155584

**Published:** 2016-05-16

**Authors:** Renwu Zhou, Rusen Zhou, Jinxing Zhuang, Zichao Zong, Xianhui Zhang, Dongping Liu, Kateryna Bazaka, Kostya Ostrikov

**Affiliations:** 1 Liaoning Key Laboratory of Optoelectronic Films & Materials, School of Physics and Materials Engineering, Dalian Nationalities University, Dalian, 116600, China; 2 School of Chemistry, Physics and Mechanical Engineering, Queensland University of Technology, Brisbane, Queensland, 4000, Australia; 3 Fujian Key Laboratory for Plasma and Magnetic Resonance, School of Physics and Mechanical & Electrical Engineering, Xiamen University, Xiamen, 361005, China; 4 Department of Chemical and Biochemical Engineering, College of Chemistry and Chemical Engineering, Xiamen University, Xiamen, 361005, China; 5 School of Information Technology and Management, University of International Business and Economics, Beijing, 10000, China; 6 CSIRO-QUT Joint Sustainable Materials and Devices Laboratory, Commonwealth Scientific and Industrial Research Organisation, P.O.Box 218, Lindfield, NSW, 2070, Australia; University Paul Sabatier, FRANCE

## Abstract

Plasma medicine is a relatively new field that investigates potential applications of cold atmospheric-pressure plasmas in bioengineering, such as for bacterial inactivation and degradation of organic molecules in water. In order to enunciate mechanisms of bacterial inactivation at molecular or atomic levels, we investigated the interaction of atmospheric-pressure air microplasmas with amino acids in aqueous solution by using high-resolution mass spectrometry (HRMS). Results show that the oxidation effect of plasma-induced species on the side chains of the amino acids can be categorized into four types, namely hydroxylation, nitration, dehydrogenation and dimerization. In addition, relative activities of amino acids resulting from plasma treatment come in descending order as follows: sulfur-containing carbon-chain amino acids > aromatic amino acids > five-membered ring amino acids > basic carbon-chain amino acids. Since amino acids are building blocks of proteins vital to the growth and reproduction of bacteria, these results provide an insight into the mechanism of bacterial inactivation by plasma.

## Introduction

Plasma applications in medical science and biological treatments have recently shown marked progress, which has received worldwide attention [1–7]. Considering that biomolecules, such as nucleic acids (RNA and DNA), amino acids, carbohydrates and lipids are essential to all known forms of life, studies of plasma interactions with these biomolecules in water at molecular or atomic levels are of fundamental importance. Reports have shown that some reactive species generated in plasma gas phase cannot penetrate the gas-liquid interface (several μm to hundreds of μm) or diffuse into the solution within their short life time during plasma treatment [8,9]. Actually, only a small portion of species, such as O_3_, H_2_O_2_, H, OH, NO_x_ and HNO_x_, can pass through the gas-liquid interface and enter the solution [10,11]. After entering the solution, some of these species exist in forms of hydrates, such as OH(H_2_O)_n_ (from OH) [12], which are present in the solution for a long time and have similar chemical properties as OH. The interaction of biomolecules in water with plasma can initiate, directly or indirectly, a variety of physical and chemical reactions. Physical conditions include the formation of UV light and shock waves, and the contribution of these factors depends strongly upon the discharge parameters [13]. Chemical conditions that occur in electrical discharges in water include the direct formation of OH, O_3_ and H_2_O_2_ among other reactive species, and the indirect formation of such species. This feature of atmospheric-pressure plasma processing is capable of facilitating certain types of chemical reactions in solution.

Although the said effects of plasma on proteins have been studied experimentally and theoretically, a definite explanation remains to be proposed about the protein inactivation mechanism at molecular or atomic levels. Lu et al. suggest that reactive species generated in the atmospheric pressure He plasma play an important role in plasmid DNA damage [14]. Kong et al. also indicate that proteins under dry conditions can be degraded by low temperature atmospheric pressure plasma (LTAP plasma) [15]. In addition, the effects of LTAP plasma on biologically functional biomolecules in aqueous solution have been reported recently [16]. LTAP plasma can degenerate amyloid-ß fibrils in solution, decrease enzymatic activity of lysozyme, increase the molecular weight of the protein, and destroy the tertiary structure of DNA. Therefore, it is relevant to further study plasma interaction at the gas-liquid interface generated during plasma exposure, which may contribute to modification and/or degradation of biomolecules in solution.

To better understand the reported changes in enzymatic activity, secondary structure, and molecular weight of proteins as a result of plasma exposure, this paper investigates the molecular and atomic level interactions of atmospheric pressure microplasmas with nine proteinogenic amino acids. Using data from mass spectroscopy, the changes in chemical structure of the said amino acids are analyzed against the plasma-generated chemical species and UV, as captured by optical emission spectroscopy, and plasma-related changes in solution temperature and pH.

## Materials and Methods

### Atmospheric-pressure Microplasma Set up

**Fig 1:**
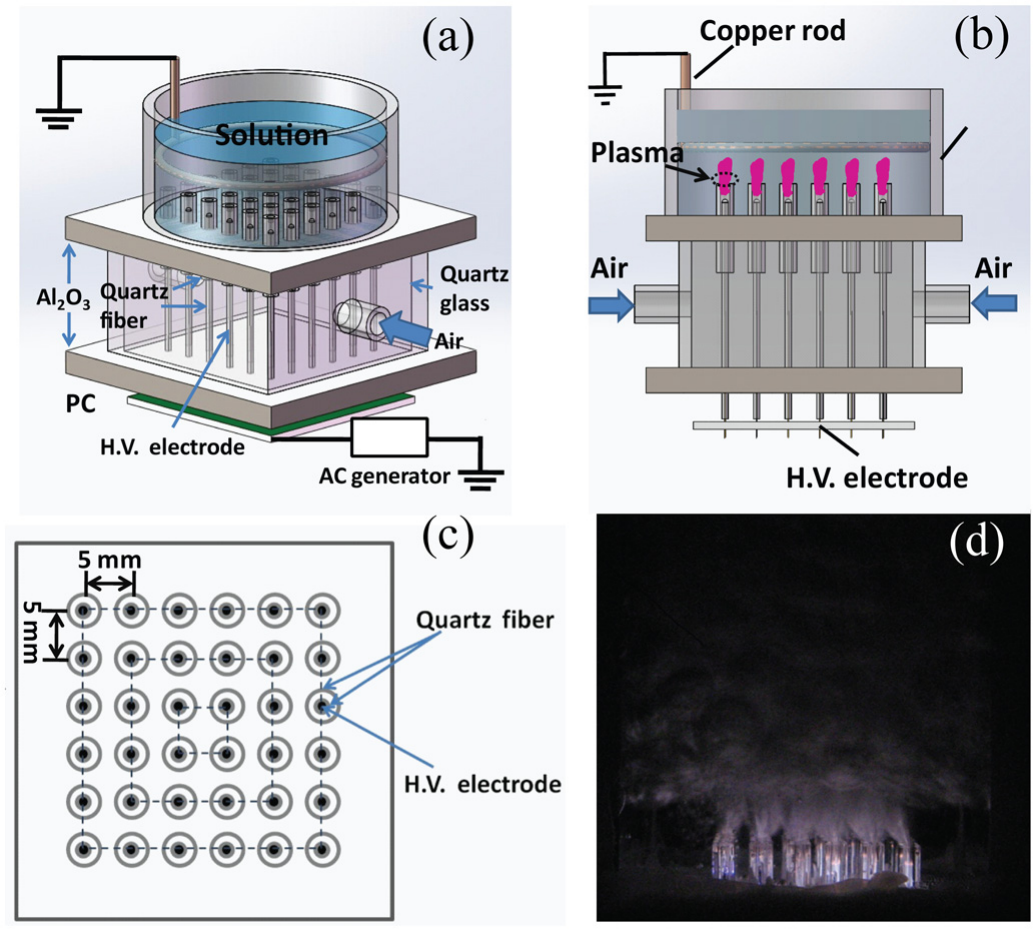
(a) Schematic diagram (3D) of the experimental setup used in this study. (b) Cross-section of the microplasma array device. (c) Top view of the plasma device. (d) photograph of electric discharge, with 20W discharge power and 2.0 SLM Air gas flow.

The schematic diagram of the custom-made microplasma array device is illustrated in Fig 1A–1C, and this atmospheric-pressure air microplasma array is used to inactivate amino acids in aqueous media (Fig 1D) [17]. Air is added into the 36 microplasma jet units at the flow rate of 4.0 standard liter per minute (SLM). The aqueous solution containing amino acids acts as the ground electrode. The power supply provides bipolar AC output with the peak voltage (V_P_) of 0–20 kV at an AC frequency of 9.0 kHz. The discharge power can be calculated by a Lissajous figure formed with the charges across the capacitor and the applied voltage across the discharge chamber. In this study, all plasma treatments of amino acids in solution are performed by using the atmospheric microplasma arrays at V_P_ = 4.0 kV, corresponding to the discharge power of 20 W.

### Chemicals and Solution Condition

L-cysteine (Cys), L-histidine (His), L-leucine (Leu), L-lysine hydrochloride (Lys), L-methionine (Met), L-phenylalanine (Phe), L-proline (Pro), L-tryptophan (Trp), L-tyrosine (Tyr) were purchased from Sangon Biotech Corp. (shanghai, China). Acetonitrile was obtained from Wako Pure Chemical Industries, Ltd (Osaka, Japan). Ultra-pure water was collected from a Milli-Q SP TOC system water purifier (Millipore Corp., Bedford, MA, USA). All amino acid samples were dissolved in ultra-pure water till the concentration reached 1 mmol/L. Then, 50 ml of amino acid solution was transferred to the discharge vessel at room temperature for plasma treatments which were performed at V_P_ = 4.0 kV within different treatment periods.

### Molecular Structure Detection

Variations in the molecular structure of amino acids in the experiment were observed using high-resolution mass spectrometry (HRMS). Mass spectra within the range of 20–500 (*m/z*) were obtained by an Orbitrap mass spectrometer (Exactive, Thermo Fisher Scientific, CA) in the electrospray ionization (ESI) positive-ion mode. The amino acid analysis was performed with an amino acid analyser (JLC-500/V2, Japan Electron Optics Laboratory). High-pressure liquid chromatography was performed on a cation ion-exchange column. The amino acids were detected by reaction with ninhydrin.

### pH Values and Temperatures of the Treated Solutions

The pH values of plasma-activated solutions were gauged with a pH meter (model Lab-850; SI Analytics Co., Mainz, Germany) as the solutions were exposed to air microplasmas. Once the plasma treatment was completed, the temperature of each plasma-treated solution was measured using a mercury thermometer.

### Optical Characteristics Analysis

Optical emission spectra (OES) from the discharge region were obtained using a SpectraPro-750i monochromator (Acton Research Corporation) with a resolution of 0.5 nm in the wavelength range of 200 to 800 nm. The rotational (T_Rot_) and vibrational (T_Vib_) temperatures of N_2_ molecules in the atmospheric-pressure air microplasmas were determined by comparing the simulated spectra of the C^3^Π→B^3^Π (Δν = 2) band transition of N_2_ with the experimental recorded spectra.

### Statistical analysis

Data sets were statistically analyzed using one-way analysis of variance (ANOVA) and linear regression analysis. *P* values below 0.05 indicated a statistically significant difference. It should be pointed out that all experiments were repeated minimum three times unless stated, and data shown represent mean ± standard error.

## Results and Discussion

### Aromatic Amino Acids (Phe and Tyr)

**Fig 2:**
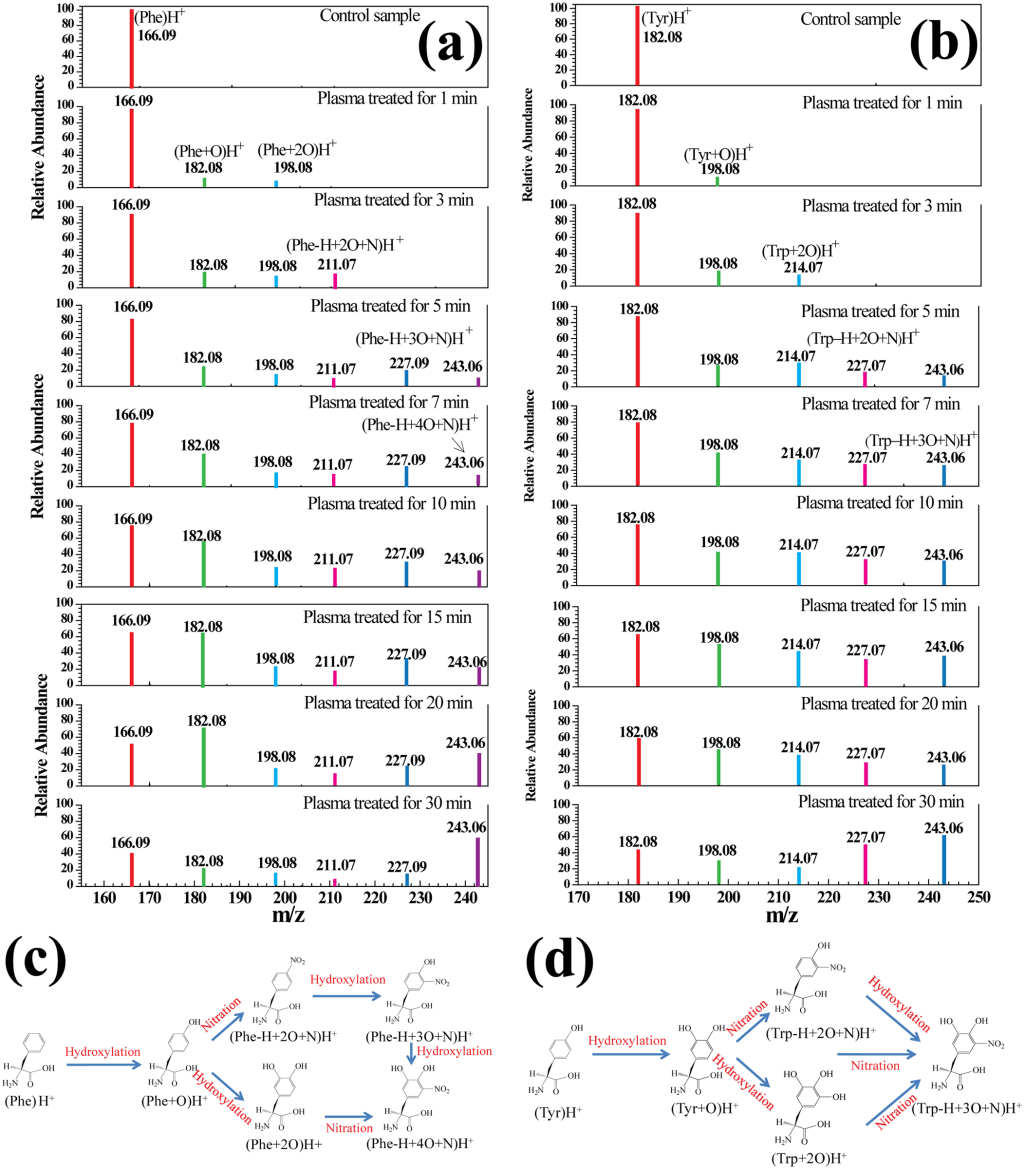
Mass spectra of aromatic amino acids Phe (a) and Tyr (b) in solution after plasma treatment for 0–30 min, and a simplified decomposition model of the aromatic amino acids Phe (c) and Tyr (d) presented according to the HRMS analysis. A molecular structure of the proposed product is shown for each peak.

Fig 2 shows the mass spectra of aromatic amino acids (Phe and Tyr) in solution after 0–30 min of plasma treatment. According to Fig 2A, (Phe)H^+^ of *m/z* 166.09 was detected in Phe solution before plasma treatment, and after the solution was treated, five oxidation products were created, namely (Phe+O)H^+^, (Phe+2O)H^+^, (Phe-H+2O+N)H^+^, (Phe-H+3O+N)H^+^ and (Phe-H+4O+N)H^+^ at *m/z* 182.08, 198.08, 211.07, 227.07and 243.06, respectively. The rapid addition of OH to the aromatic ring (Phe) with little selectivity yielded a hydroxyl cyclohexadienyl radical. Further hydroxylation produced DOPA and TOPA. Similarly, Fig 2B shows that (Tyr)H^+^ of m/z 182.08 was detected in a solution of Tyr, and the four oxidation products of plasma treatment were (Tyr+O)H^+^ at *m/z* 198.08, (Tyr+2O)H^+^ at *m/z* 214.07, (Tyr-H+2O+N)H^+^ at *m/z* 227.07, and (Tyr-H+3O+N)H^+^ at *m/z* 243.06. OH can be added to the sites adjacent to the original hydroxyl at aromatic side chains (Tyr), leading to multiple hydroxylation. Previous studies have proved that plasma treatment of Phe and Tyr solutions contributes to the formation of various oxidation products, including 1, 2, and 3 oxygen atoms containing nitryl [18], which is consistent with our experiment data. A simplified decomposition model of the aromatic amino acids is presented in Fig 2C and 2D, according to the HRMS analysis mentioned above. These oxidation products are presumed to arise as a result of amino acids being subjected to attack by energetic electrons, followed by their interactions with various radicals, such as hydroxyl and nitro groups. During the plasma treatment, OH groups replaced the H atoms attached to one or several C atoms in the benzene ring, which contributed to the formation of phenols, including (Phe+O)H^+^, (Phe+2O)H^+^, (Tyr+O)H^+^ and (Tyr+2O)H^+^. With the increase in treatment time, the bond between OH and the benzene ring was destroyed, and OH groups were further oxidized and replaced by -NO_2_ groups. These results suggest that the benzene ring of aromatic amino acids could be easily hydroxylated and nitrated by the ROS and RNS generated in plasma. The phenol functionality plays an important role in signal transduction, where it performs as a receiver of phosphate groups facilitated by receptor tyrosine kinases. Phosphorylation of the OH group of the phenol changes the enzymatic activity of the target protein in response to growth factors, cytokines, and hormones, and as such is a key regulator of normal cellular processes. Many antibiotics target the shikimate pathway The g UV of VUV/aster in attaining the same level of treatment outcomes as ROS-only, suggesting important photochemical reactiinvolved in aromatic acid synthesis in bacterial cells.

### Five-Membered Ring Amino Acids (His and Pro)

**Fig 3:**
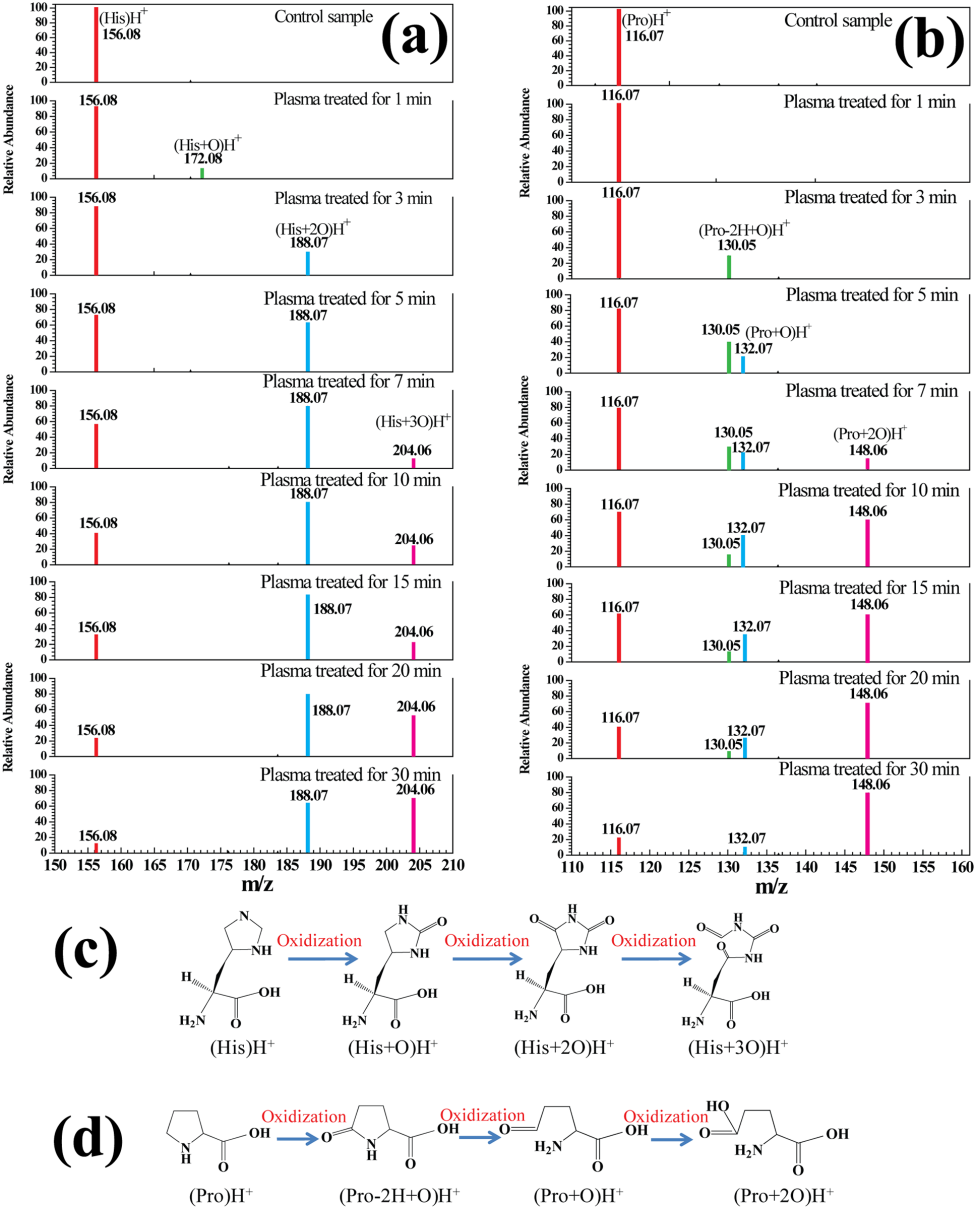
Mass spectra of five-membered ring amino acids His (a) and Pro (b) in solution after plasma treatment for 0–30 min, and a simplified decomposition model of the five-membered ring amino acids His (c) and Pro (d) presented according to the HRMS analysis. A molecular structure of the proposed product is shown for each peak.

Fig 3 shows the mass spectra of five-membered ring amino acids (His and Pro) in solution after 0 to 30-min plasma treatment. Fig 3A shows that (His)H^+^ was detected in His solution at *m/z* of 156.08 before plasma treatment, and the mass spectrum of the plasma-treated His solution gives information about three oxidation products, i.e. (His+O)H^+^, (His+2O)H^+^ and (His+3O)H^+^ at m/z 172.08, 188.07, and 204.06 respectively. It could be seen from Fig 3C that His was initially oxidized to (His+O) by plasma treatment and then rapidly oxidized to (His+2O)H^+^ which, given enough plasma treatment time, transformed into a ring-opened structure (His+3O)H^+^. This means that plasma treatment might contribute to ring-opening of His. Fig 3B shows that (Pro)H+ at m/z 116.07 was converted into three oxidation products prior to plasma treatment, namely (Pro-2H+O)H^+^ at m/z 130.05, (Pro+O)H^+^ at m/z 132.07 and (Pro+2O)H^+^ at m/z 148.06. Fig 3D shows that the -CH_2_ groups in the five-membered ring of Pro could be readily oxidized to -C = O groups. Furthermore, the -NH-C = O bond could be broken so that the five-membered ring was opened for the formation of ketone [19], which could be further oxidized to carboxylic acid. These results indicate that five-membered ring amino acids could be ring-opened more easily by various radicals than could aromatic amino acids.

### Sulfur-Containing Carbon-Chain Amino Acids (Cys and Met)

**Fig 4:**
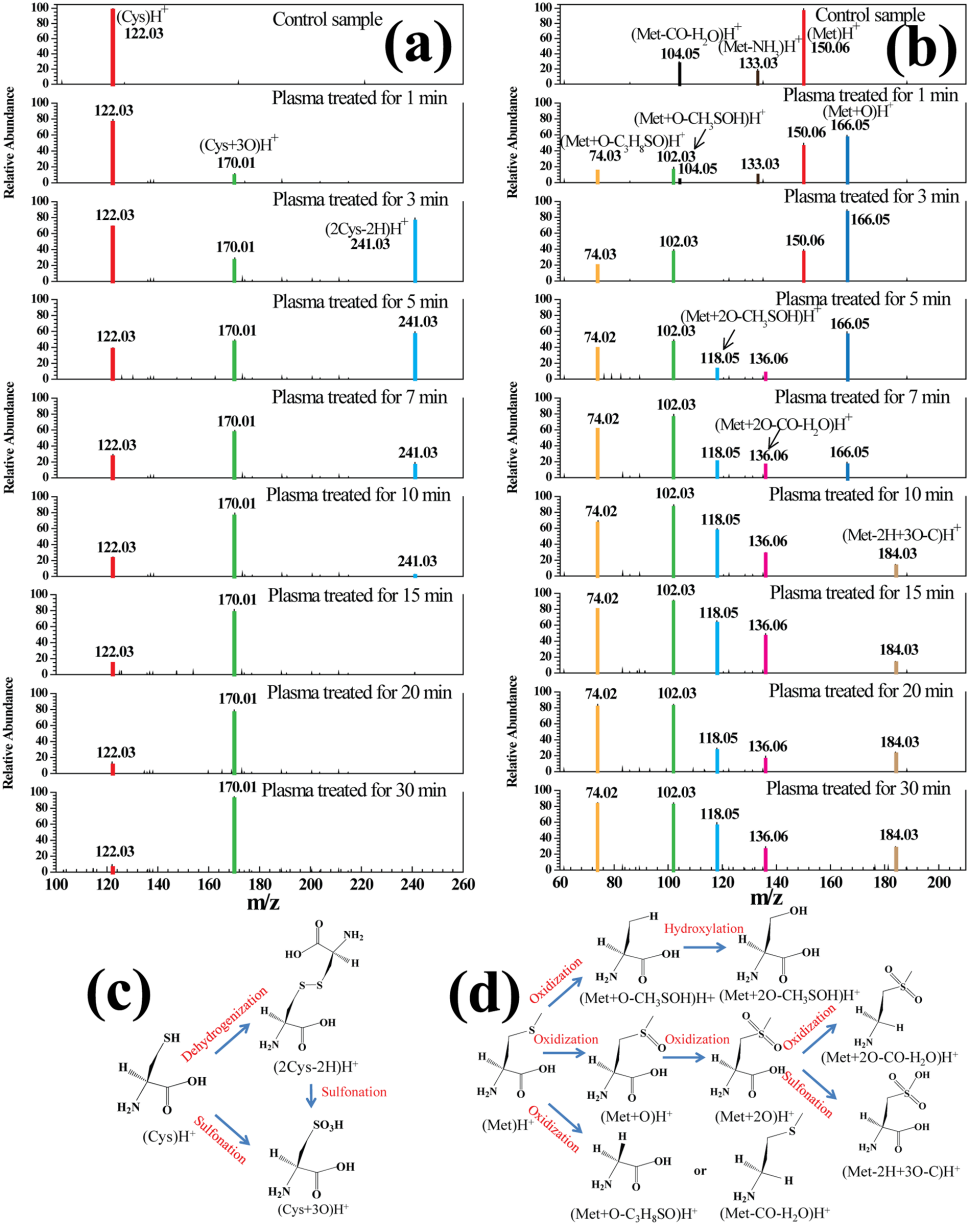
Mass spectra of sulfur-containing carbon-chain amino acids Cys (a) and Met (b) in solution after plasma treatment for 0–30 min, and a simplified decomposition model of Cys (c) and Met (d) presented according to the HRMS analysis. A molecular structure of the proposed product is shown for each peak.

Fig 4 shows the mass spectra of sulfur-containing carbon-chain amino acids (Cys and Met) in solution after 0–30 min of plasma treatment. In the control Cys solution, (Cys)H^+^ at m/z 122.03 was detected as shown in Fig 4A, and the mass spectrum of Cys after plasma treatment displayed two oxidation products—(Cys+3O)H^+^ and (2Cys-2H)H^+^ at m/z 170.01 and 241.03, respectively. It has been made clear in literature that among amino acids, Cys had the most accessible H-abstraction because the average single bond energies for S-H, O-H, N-H, and C-H stand at 363, 459, 386, and 411 kJ/mol respectively, at 25°C. Experiment results indicate that highly reactive thiol group–SH of Cys branched chain was easily dehydrogenized, resulting in the formation of (2Cys-2H)H^+^, which could be further oxidized to sulfonic group–SO_3_ [20]. Fig 4B shows that (Met)H^+^, (Met-CO-H_2_O)H^+^ and (Met-NH_3_)H^+^ at m/z 150.6, 104.05 and 133.03 were present before the plasma treatment. After 5 min of plasma treatment, (Met)H^+^, (Met-CO-H_2_O)H^+^ and (Met-NH_3_)H^+^ were no longer observed in the mass spectrum, and (Met+O-C_3_H_8_SO)H^+^ at m/z 74.02, (Met+O-CH_3_SOH)H^+^ at m/z 118.05 and (Met+O)H^+^ at m/z 166.05 were produced by oxidization instead. Met was highly reactive with the OH radical and the reaction resulted in different intermediate radical species [21]. Illustrated in Fig 4C and 4D are the main sequential steps involved in the treatment of sulfur-containing carbon-chain amino acids by the air microplasma. These results show that sulfur-containing carbon-chain amino acids could be rapidly oxidized and sulfonated as a result of exposure to ROS.

### Carbon-Chain Amino Acids (Leu and Val)

**Fig 5:**
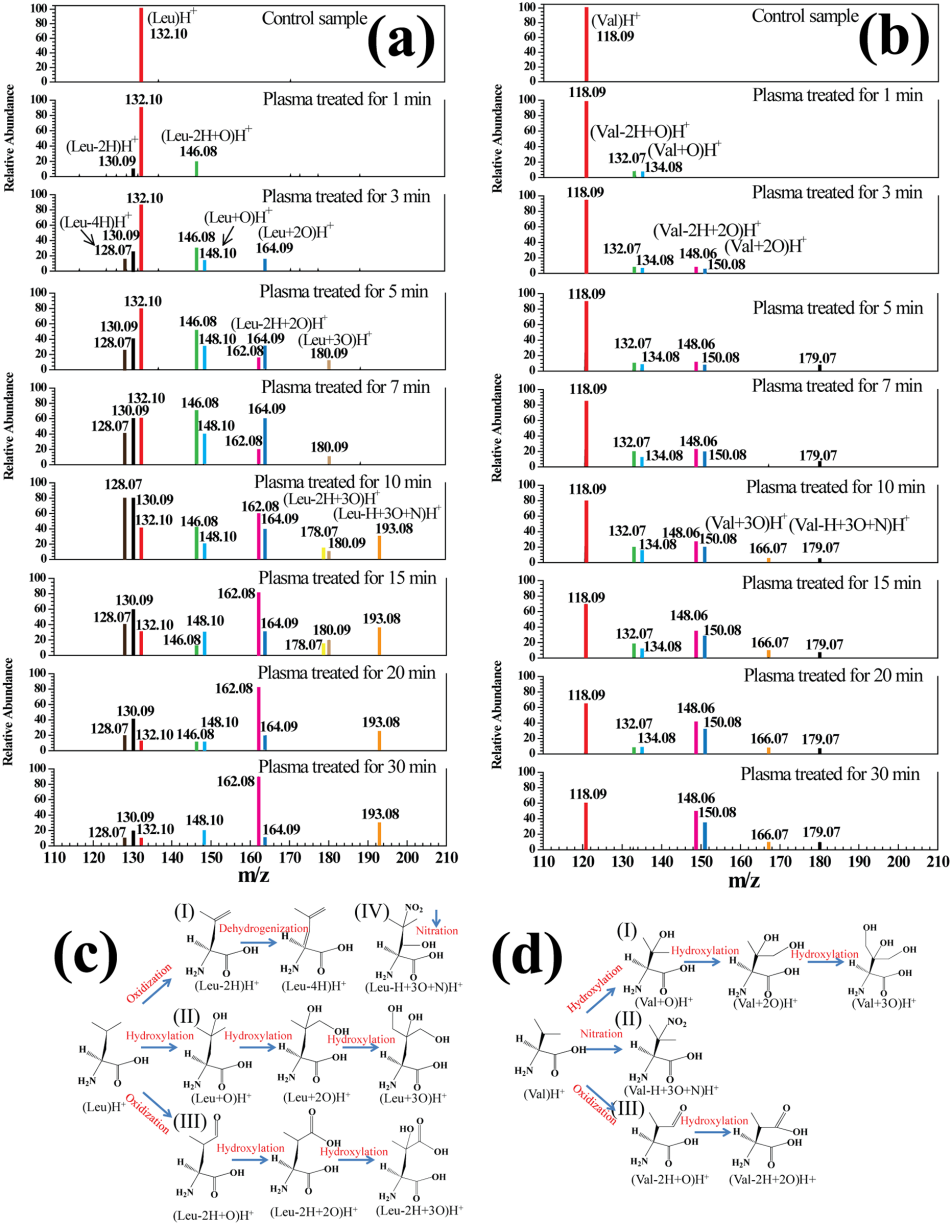
Mass spectra of carbon-chain amino acids Leu (a) and Val (b) in solution after plasma treatment for 0–30 min, and a simplified decomposition model of Leu (c) and Val (d) presented according to the HRMS analysis. A molecular structure of the proposed product is shown for each peak.

Fig 5 shows the mass spectra of carbon-chain amino acids (Leu and Val) in solution after plasma treatment for 0–30 min and at V_P_ = 4.0 kV. In the Leu solution prior to plasma treatment, (Leu)H^+^ was detected at m/z 132.10 (See Fig 5A). Various products were decomposed and oxidized in four pathways in the Leu solution after plasma treatment (See Fig 5C). Pathway I: Leu molecules could be dehydrogenated and converted into (Leu-2H)H^+^ and (Leu-4H)H^+^ at m/z 130.09 and 128.07 by plasma. Pathway II: OH radicals in solution could attack Leu molecules, resulting in the formation of (Leu+O)H^+^, (Leu+2O)H^+^ and (Leu+3O)H^+^ at m/z 148.10, 164.09 and 180.09 by hydroxylation. Pathway III: formylation, carboxylation and hydroxylation-carboxylation of (Leu-2H+O)H^+^, (Leu-2H+2O)H^+^ and (Leu-2H+3O)H^+^ were observed in ROS oxidation reactions. Pathway IV: (Leu-H+3O+N)H^+^ at m/z 193.8 was hydroxylated-nitrated by RNS in plasma. Similarly, (Val)H^+^at m/z 118.09 was detected before plasma treatment (See Fig 5B). During the plasma treatment, six new substances were detected at m/z 132.07, 134.08, 148.06, 150.08, 166.07 and 179.07 respectively. The detailed reaction process was depicted in Fig 5D. (I) (Val)H^+^ was hydroxylated by OH radicals to (Val+O)H^+^, which could be further di-hydroxylated or tri-hydroxylated to (Val+2O)H^+^ or (Val+3O)H^+^. (II) A trace of (Val)H^+^ could be firstly hydroxylated and then nitrated into (Val-H+3O+N)H^+^ by OH and NO_x_ radicals. (III) (Val)H^+^ was formylated to (Val-2H+O)H^+^ which could be further carboxylated into (Val-2H+2O)H^+^ by oxygen-containing species. OH radicals attacked the aliphatic hydrocarbon side chains (e.g., Val, and Leu) indiscriminately, and as the number of C-H bonds and the length of the hydrocarbon side chains increased, the reactivity improved [21,22]. Various products were observed after plasma treatment of carbon-chain amino acids largely because of the formation of unsaturated bonds.

### Relative Reactivity of Amino Acids to Plasma Treatment

**Fig 6:**
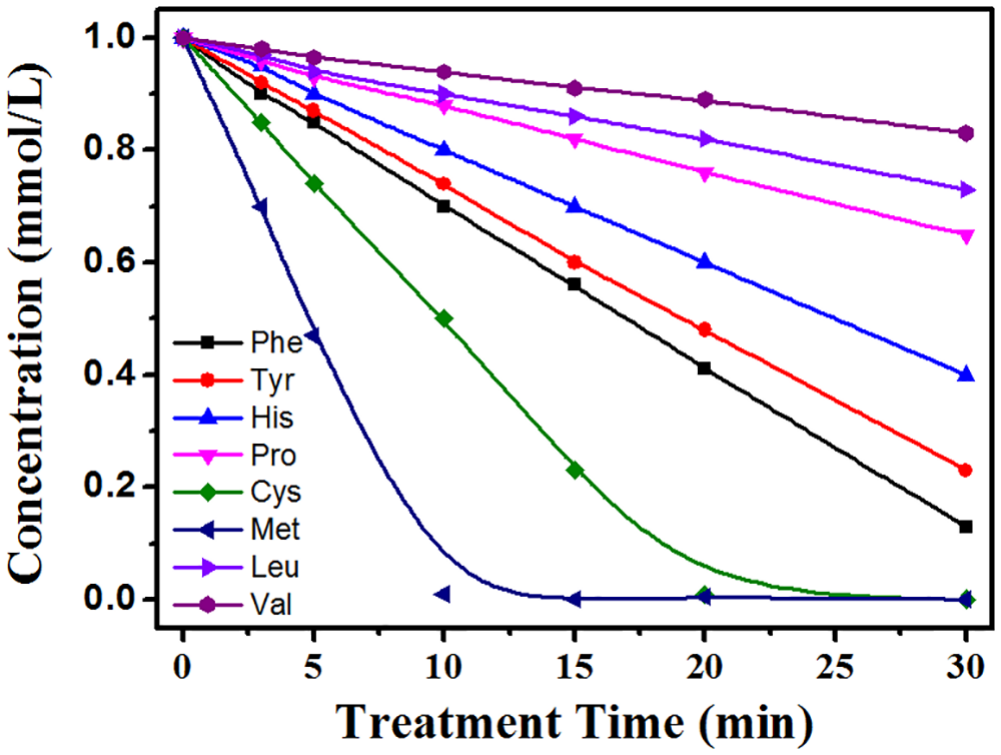
Relative reactivity of amino acids due to plasma treatment estimated from a competitive experiment.

The four types of aforementioned amino acids were mixed in aqueous solution and subjected to microplasma treatment for the indicated time. From the competitive experiment, relative reactivities of amino acids to plasma treatment were estimated. Fig 6 shows that the reactivity of the 8 amino acids descended in the following sequence: Met > Cys > Trp > Phe > Tyr > His > others. Notably, Met was completely degraded after 10 min of treatment. Thus, relative reactivities of amino acids to plasma treatment most likely go as follows: sulfur-containing carbon-chain amino acids being the most reactive, followed by aromatic amino acids, five-membered ring amino acids, basic carbon-chain amino acids listed in order of decreasing reactivity.

### pH value of the treated solution

**Fig 7:**
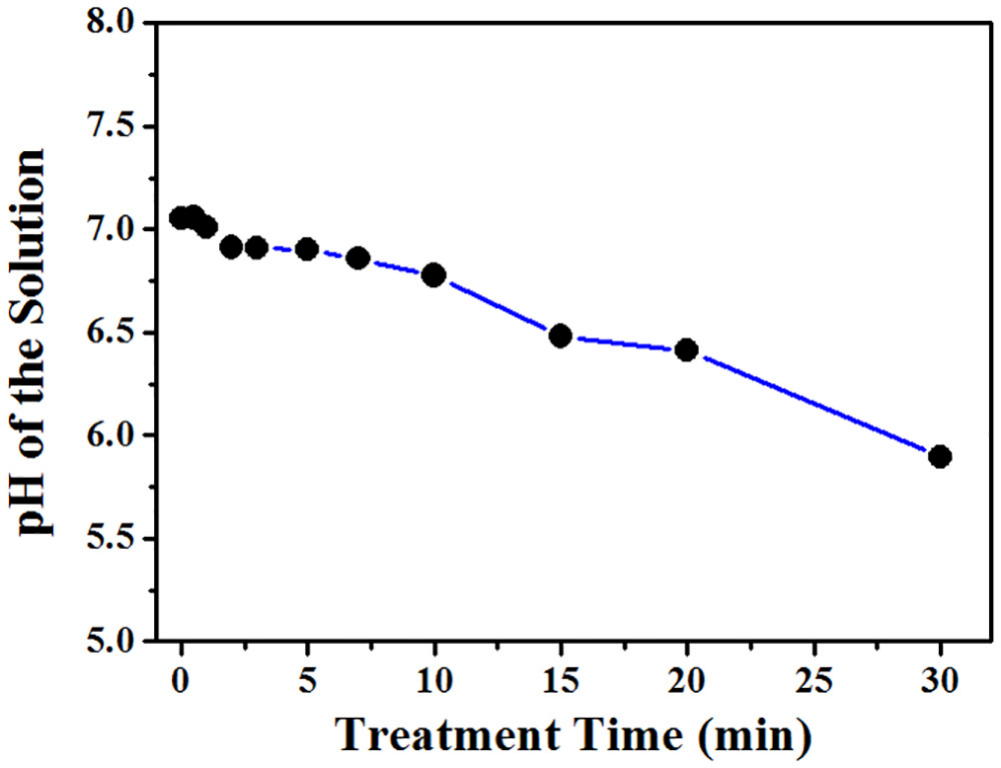
The pH value of the treated solution as a function of treatment time.

Acidification is one of the most biologically significant effects that plasma treatment may have on an aqueous solution. A significant decrease in solution pH has been reported by several studies [23–25]. Fig 7 shows the pH values of the mixed solution containing different amino acids at different treatment time. Results show that the atmospheric-pressure air microplasma arrays resulted in a slight decrease in the pH value (5.247 after air plasma treatment for 30min). This might be due to the NO_X_ produced in the plasma reacting with water and producing nitric and nitrate acids [26]. Plasma-induced species, including radicals and charged species (electrons and ions) have been found by many to play the role in the modification of biomolecules by low temperature plasmas [27,28]. In our experiment, the aqueous solution containing amino acids was subject to all possible agents generated in the microplasma jets, including various short-lived radicals and ions. The extent (or the nature) of chemical modifications of amino acids as a result of plasma treatment observed in this study suggests the chemical reactions of amino acids with plasma-generated reactive species as the primary mechanism of amino acid degradation, with some contribution from chemical degradation by acidic pH [29, 30].

### OES spectra of microplasma array

**Fig 8:**
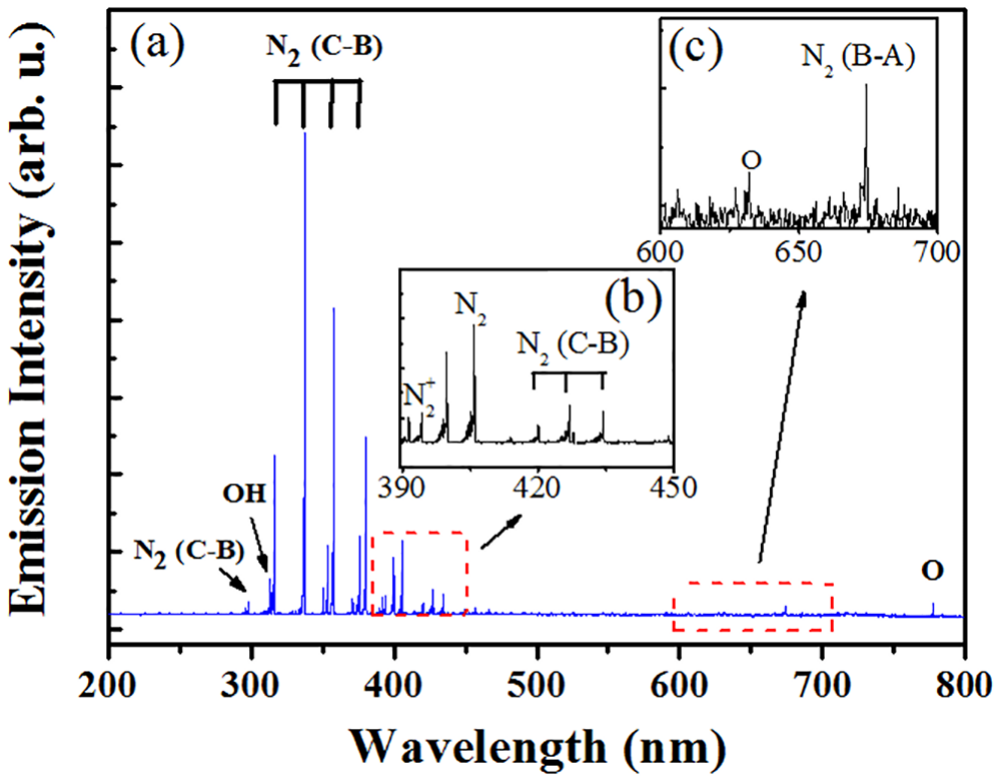
The emission spectrum of the air microplasma array for (a) 200–800 nm. The insets (b) and (c) show the extended emission spectra.

Fig 8A shows the typical OES spectra of the air microplasma array generated at a V_P_ of 4.0 kV. The insets (Fig 8B and 8C) show the extended emission spectra. This spectrum was dominated by N_2_ (C→B) emissions as a result of many excitation processes such as electron impact excitation from the molecular ground state N_2_ (X^1^_g_^+^) and first metastable state N_2_ (A^3^_u_^+^), pooling reaction, and transfer of energy in collisional relaxation processes [31]. The O and OH emission lines were also clearly observed at 777 nm and 309 nm respectively, which are presumably due to the direct electronic impact dissociations of O_2_ and H_2_O molecules [32]. Plasma-induced metastable or excited species can emit UV photons, which along with reactive species and ions contribute to effective decontamination and degradation of organic macromolecules and living cells often observed in plasmas [33,34].

Indeed, exposure to strong UV radiation can lead to significant damage of amino acids, including photo-oxidative breakdown or cross-linking. For plasma-enabled degradation, in most instances it is very difficult to discern respective contributions of photons and reactive species [35]. Furthermore, a potential significant contribution to biological activity of plasmas may come from the synergistic effects that arise from simulatanous exposure of the treated substance or organism to photons and chemical species. Using a modified plasma jet device capable of separation of UV photons and heavy reactive species in the effluent, Schne ider et al. demonstrated that effect of UV treatment was limited, whereas the effect of ROS-only treatment was consistent with expected dose-dependent behaviour [35]. The combined UV/ROS treatment was considerably faster in attaining the same level of treatment outcomes as ROS-only, suggesting important photochemical reactions that potentially facilitiate the treatment by allowing more reactive or excited species to reach the target. The photooxidation of such amino acids as Cys, His, Met, Tyr, and Try is well understood and can occur via type I and/or type II (^1^O_2_) photooxidation pathways. For example, photochemical degradation of Cys and Met involves both type I and type II reactions, whereas His primarily by ^1^O_2_, and Try predominantry by type I with some ^1^O_2_ reactions [34]. The photooxidation of Tyr also undergo photochemical degradation by both type I and type II reactions, with the latter being highly pH dependent [36]. The emission spectra obtained by high-sensitive UV detector indicates that some significant UV emission from these microplasma jets occurred in the 200–400 nm wavelength range. UV C (100–280nm) is obviously the most destructive for organic molecules, then UV B (280–315nm), with UV A (315–400nm) being least damaging. Specially, OH radicals emitted at 309 nm should be most effective to drive chemical reactions with amino acids due to their high oxidizabilities. Jablonowski et al. also verified the photochemical contribution of plasma jet-emitted VUV radiation in the formation of hydroxyl radicals in the liquid via dissociation of water [37].

### Temperature of the microplasma and treated solution

**Fig 9:**
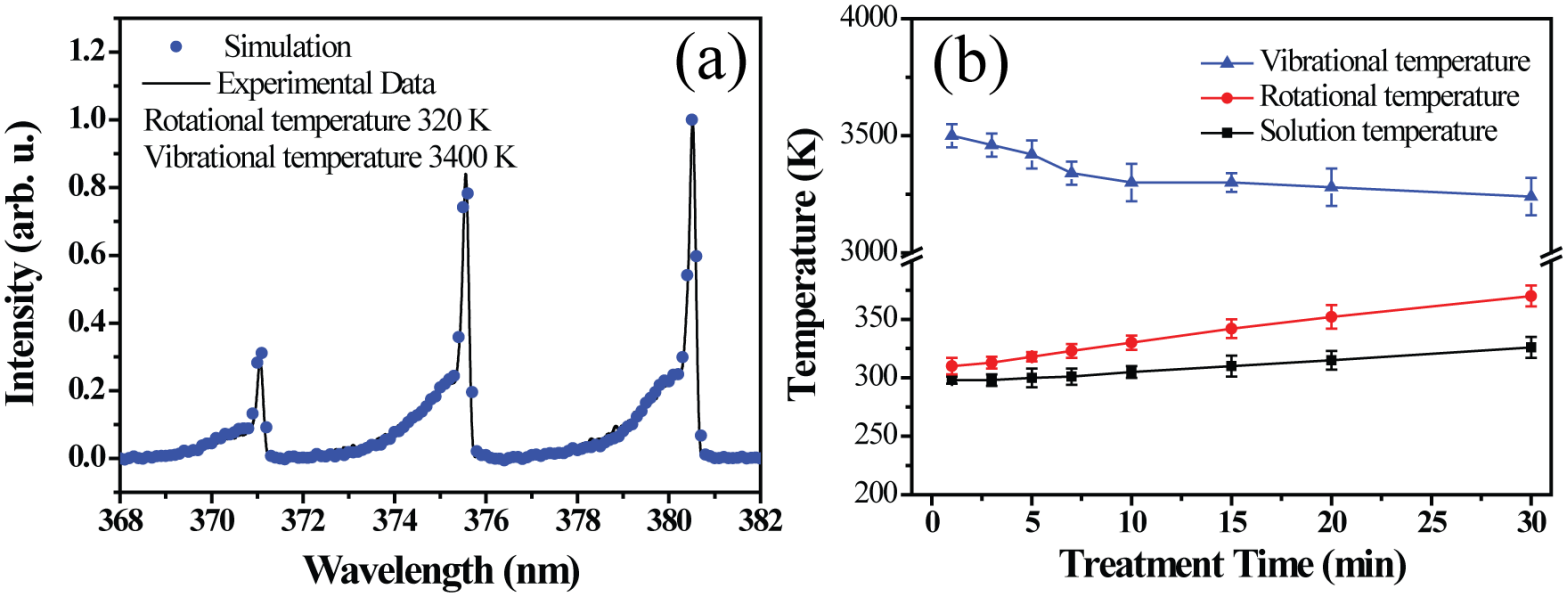
(a) The comparison between the measured and calculated emission spectra of N_2_ second positive system. (b) T_vib_, T_Rot_ values of the N_2_ (C^3^Π_u_) state and the temperature of treated solution as a function of treatment time.

Optical emission spectra (OES) of the second positive bands of N_2_ (C^3^Π_u_→B^3^Π_g_) were recorded to calculate the rotational temperature by comparing the experimental spectra with the calculated ones, and the gas temperature was obtained with the best fitted spectra using the Specair code, as shown in Fig 9A. This fitting led to T_Rot_ = 320±40 K and T_Vib_ = 3400±100 K. In addition, both the rotational and vibrational temperatures were plotted as a function of the plasma treatment time. The rotational and vibrational temperature were typically in the range between 310–370 K and 3240–3500K, and the temperature of the treated solution obtained by the infrared thermometer was in range between 298 and 326 K, which is a little lower than the rotational temperature, as shown in Fig 9B. This difference may be caused by the loss of energy transformation in the aqueous solution. Results indicate that the slight increase in water temperature does not cause an obvious effect on the modification of amino acids.

### Effect of Plasma-Generated Oxygen Species on Amino Acids

Plasma generated in solution induces chemical reaction not only on the surface of the liquid but also within the liquid because of the diffusion of ROS generated in the gas phase. The generation of these new products is attributed to the large number of active species formed in the air-discharge process, including (1) negative particles (e^-^, O^-^ and O_2_^-^); (2) positive particles N_2_^+^, N_4_^+^, O^+^, O_2_^+^, O_4_^+^, H^+^, H_2_^+^, OH^+^, NO^+^ and NO_2_^+^; (3) atoms N, O and H; and (4) molecules N_2_, O_2_, O_3_, OH, HO_2_ and NO_x_. The processes involved in OH formation are: e + O_2_→ e + O(^3^P) + O(^1^D) [8] and O(^1^D) + H_2_O → 2OH (X^2^Π_2/3_) [10]. The OH is in X^2^Π_2/3_ state. Otherwise short-lived in the aqueous solution, some OH radicals are converted into H_2_O_2_ and OH(H_2_O_2_)_n_, which can exist in water for a long time. In addition, two reaction processes of H_2_O_2_ occur in water, namely (1) the reversible reaction: H_2_O_2_ ↔ H^+^ + HO_2_^-^ and (2) the decomposition reaction under UV light irradiation: H_2_O_2_→2OH. Thus, the increase in H_2_O_2_ content brings about the increase in OH radicals and HO_2_^-^ ions. Aside from OH, many ozone molecules are generated in water by plasma (as evidenced by the pungent smell). The reaction processes include O(^1^D) + O_2_→O(^3^P)+ O_2_(b^1^∑g^+^)→O(^3^P) + O_2_(a^1^Δ)→O(^3^P)+ O_2_(a^3^∑u^+^) and O(^3^P) + O_2_(a^3^∑u^+^) + O_2_ → O_3_+ O_2_(a^3^∑u^+^). While being dissolved, some ozone molecules can directly react with water, and then decompose to O and O_2_. The reaction process is O_3_ + H_2_O →2OH + O_2_ under UV irradiation. The O molecule in turn reacts with H_2_O to generate more OH. Finally, the NO_x_ generation can be described by the following formulas: e + N_2_→N + N + e, N + O + N_2_→ NO + N_2_, NO + O_3_ → NO_2_ + O_2_, NO_2_ + H_2_O → HNO_2_(aq) + HNO_3_(aq) [17,38].

### Effect of Plasma-Generated OH Radicals on Amino Acids

Generally, OH radicals react swiftly and indiscriminately with most electron-rich sites of organic molecules [21]. The reaction was initiated through hydroxyl addition to the C = C bonds or aromatic rings or through hydrogen abstraction from saturated carbon sites of molecules. These reactions create transitory radical species, which go through further reactions subject to the radical and structural environment [19]. The determining factors for both types of reactions include the possible number of sites accessible to OH radicals, the electronegativity of the substitutes on the target sites, the intensity of the C-H bond, the steric effects, and the property of the produced organo radical [39]. Based on these factors and experimental data, we can conclude that (1) for aromatic amino acids, the rapid addition of OH to the aromatic ring (Phe or Tyr) with little selectivity yielded a hydroxyl cyclohexadienyl radical; (2) for five-membered ring amino acids, a large number of products were formed in the His and OH reaction yet some oxidation products have not been fully identified [22,40]; the five-membered ring amino acids could be ring-opened more easily by OH radicals than could aromatic amino acids [41]. (3) for sulfur-containing carbon-chain amino acids, sulfur-containing side chains with high reactive thiol group -SH are susceptible to dehydrogenization and oxidization by OH radicals; and (4) for carbon-chain amino acids, a wide range of products can be obtained after plasma treatment owing to the formation of unsaturated bonds by OH radicals.

### Effect of Plasma-Generated Ozone on Amino Acids

Ozone is known to be unstable in aqueous solution and decomposable through several initiation, propagation, and termination chain reactions [42,43]. O_3_ could react with various inorganic and organic compounds at different rates [44]. Specifically, the rate constants for a wide variety of amino acids, except Cys, Met, and Trp, when reacting with plasma, ranged from 2.6 × 10^4^ M/s to 4.4 × 10^6^ M/s [45]. The reactivity was presented in ascending order: Val < Leu < Pro <His < Tyr < Phe. Significantly, the high reactivity of Cys and Met with O_3_ suggests that the possible site of reaction was at the sulfhydryl functional group rather than the amino group. It is likely that the imidazole ring in His and the five-membered ring in Pro as a secondary amine contributed to the high reactivity of these two amino acids with O_3_. As for aromatic amino acids, Phe and Tyr, high reactivity with O_3_ was also observed, which reflects that the aromatic ring and the benzylic hydrogens might influence the reactivity with O_3_. However, hydrocarbon-like amino acids (Val and Leu) have side chains that showed low reactivity with O_3_.

### Effect of Plasma-Generated H_2_O_2_ on Amino Acids

Compared with other radicals, H_2_O_2_ can exist in the solution for a longer period of time and over a wider range. The oxidation of H_2_O_2_ is attributed to the increase in the content of OH, HO_2_^−^ radicals and O_2_^•−^ in the solution, which is proportional to the concentration of H_2_O_2_ (H_2_O_2_ + *hν* → 2OH, H_2_O_2_ + OH →HO_2_•+ H_2_O, HO_2_^−^ + OH → O_2_^•−^ + H_2_O). It is widely known that O_2_^•−^ reacts with alkyl sulfide via nucleophilic substitution or acts as a moderate one-electron reducing agent on large numbers of transitional metal compositions and organic compounds [46,47]. On the other hand, the reactivity of O_2_^•−^ was remarkably lower than that of other ROS (by several orders of magnitude); HO_2_^**•**^ also serves as a weak oxidizer while the OH radical was the most reactive oxidant with practically diffusion-controlled rate constants.

## Conclusion

In this study, the effects of plasma treatment on chemical structures of amino acids which constitute proteins were investigated by HRMS. Based on the experiment results, a rough picture of the interaction mechanism of amino acids and air plasma could be drawn. Aromatic amino acids could be hydroxylated and nitrated, while five-membered-ring amino acids could be oxidized and ring-opened. Moreover, sulfur-containing carbon-chain amino acids were relatively active and could be sulfonated by ROS quickly. Carbon-chain amino acids could be oxidized to a variety of oxidized products through dehydrogenation, hydroxylation, nitration and dehydrogenation. The results obtained in this study provide a crucial first step for understanding of plasma interactions with biomolecules at molecular or atomic levels and have implications for the potential use of plasma in biology and water sterilization.
